# Feasibility of Remote Real-Time Guidance of a Cardiac Examination Performed by Novices Using a Pocket-Sized Ultrasound Device

**DOI:** 10.1155/2013/627230

**Published:** 2013-08-20

**Authors:** Tuan V. Mai, David T. Ahn, Colin T. Phillips, Donna L. Agan, Bruce J. Kimura

**Affiliations:** Department of Graduate Medical Education, Scripps Mercy Hospital, 4077 Fifth Avenue, MER-35, San Diego, CA 92103, USA

## Abstract

*Background*. The potential of pocket-sized ultrasound devices (PUDs) to improve global healthcare delivery is limited by the lack of a suitable imaging protocol and trained users. Therefore, we investigated the feasibility of performing a brief, evidence-based cardiac limited ultrasound exam (CLUE) through wireless guidance of novice users. *Methods*. Three trainees applied PUDs on 27 subjects while directed by an off-site cardiologist to obtain a CLUE to screen for LV systolic dysfunction (LVSD), LA enlargement (LAE), ultrasound lung comets (ULC+), and elevated CVP (eCVP). Real-time remote audiovisual guidance and interpretation by the cardiologist were performed using the iPhone 4/iPod (FaceTime, Apple, Inc.) attached to the PUD and transmitted data wirelessly. Accuracy and technical quality of transmitted images were compared to on-site, gold-standard echo thresholds. *Results*. Novice versus sonographer imaging yielded technically adequate views in 122/135 (90%) versus 130/135 (96%) (*P* < 0.05). CLUE's combined SN, SP, and ACC were 0.67, 0.96, and 0.90. Technical adequacy (%) and accuracy for each abnormality (*n*) were LVSD (85%, 0.93, *n* = 5), LAE (89%, 0.74, *n* = 16), ULC+ (100%, 0.94, *n* = 5), and eCVP (78%, 0.91, *n* = 1). *Conclusion*. A novice can perform the CLUE using PUD when wirelessly guided by an expert. This method could facilitate PUD use for off-site bedside medical decision making and triaging of patients.

## 1. Background

Although pocket-sized ultrasound devices (PUDs) can augment the physical exam, their potential to improve global healthcare delivery in the emergent setting or in areas of limited resources has been limited by the lack of a suitable imaging protocol and trained users [1]. We have previously shown that an evidence-based, 5-minute “quick-look” cardiac limited ultrasound exam (CLUE) has diagnostic and prognostic value [2], affects medical decision making [3], and can be taught to internal medicine residents [4]. Unlike the traditional bedside cardiac examination, CLUE has the potential for wireless transmission. Recent reports have shown the feasibility of remotely mentored ultrasound examination to evaluate apnea and pneumothorax in trauma [5] and in off-line remote expert echo interpretation using a smartphone-based application [6]. We investigated the feasibility of utilizing low-cost, readily available wireless video conferencing software to guide novice users in performing CLUE in real time.

## 2. Methods

The CLUE examined 4 targets using 4 previously-validated, nonquantitative ultrasound “signs” [2]. In brief, the parasternal long axis view was assessed for LV systolic dysfunction (LVSD) and left atrial enlargement (LAE). LVSD was present if the anterior leaflet of the mitral valve during diastole did not appear to encroach upon the LV outflow tract and approach the septum to within approximately 1 cm by nonquantitative estimation. LAE was present if the anteroposterior diameter of the LA appeared larger than that of the overlying aorta throughout the cardiac cycle. Longitudinal views at the mid-infraclavicular intercostal space of each lung apex were assessed for extravascular lung water by ultrasound lung comet-tail artifacts (ULC+) [7], considered present when 3 vertical hyperechoic lines per image were seen to emanate from the pleural line in the near field and reach the far field, in either lung apical view. Each lung apex was a separate CLUE view and a positive ULC sign was defined when either lung apex or both lung apices were ULC+. The subcostal longitudinal view of the proximal intrahepatic inferior vena cava as it entered the right atrium, within approximately 1-2 cm of the diaphragm, was assessed for elevated central venous pressures (eCVP), defined when the IVC appeared plethoric by visual interpretation with parallel vessel walls and a luminal diameter reduction of <50% with respiratory motion of the diaphragm, without forced “sniffing” (see Table 1).

**Figure 1:**
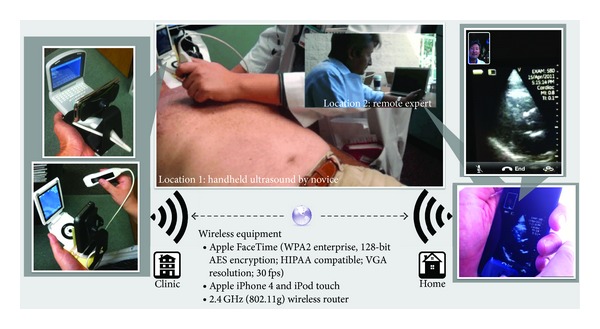


**Figure 2:**
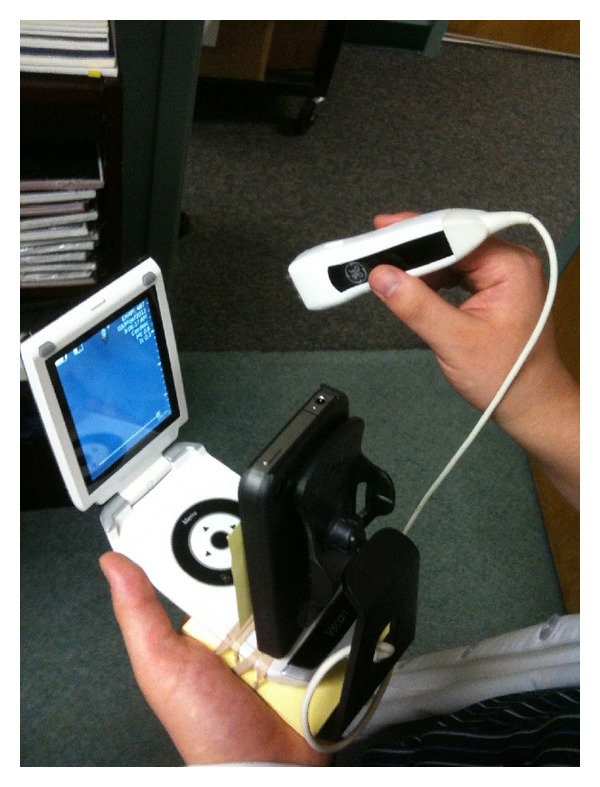


**Figure 3:**
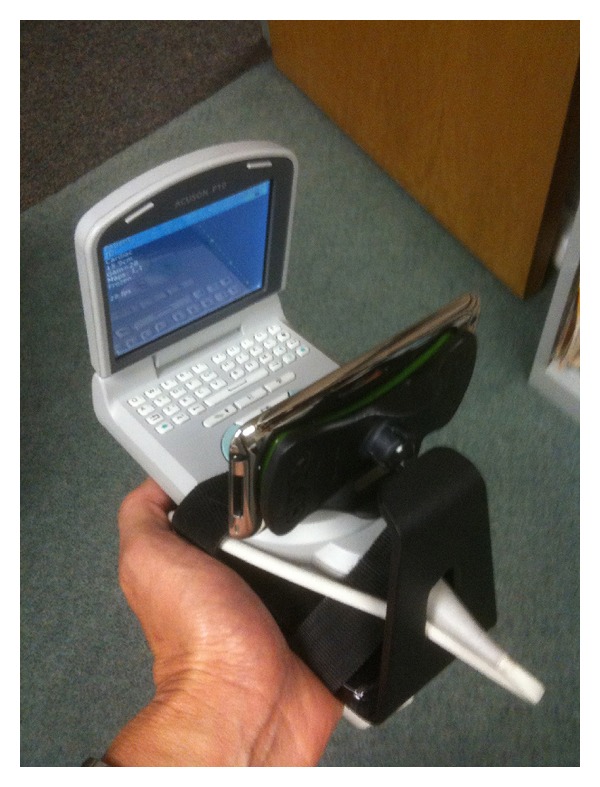


Three trainees (medical student, intern, and pharmacy resident) with less than 1-hour device orientation and no prior ultrasound experience or training performed the CLUE using a PUD (either the Vscan, GE Healthcare, or the P10, Siemens Healthcare) on 27 subjects (22 outpatients and 5 normal volunteers) at a medical office while being directed by an off-site cardiologist through an iPhone 4 or iPod (Apple Inc.) attached to the PUD as follows. The iPhone or iPod had been affixed to the base of the PUD using a small commercially available dashboard phone mount so that the iPhone/iPod's front facing 0.3 megapixel VGA camera was directed at the opposing PUD display screen (see Figures 1, 2, and 3). Using the application Apple FaceTime (WPA2 Enterprise, 128-bit AES Encryption; HIPAA Compatible; VGA resolution; 30 fps), data was transmitted wirelessly through currently available off-the-shelf Wi-Fi networks (2.4 GHz, 802.11 g, wireless router) to the cardiologist's receiving iPod. The cardiologist provided remote audiovisual guidance with real time feedback on operation of the PUD, image acquisition, and interpretation for CLUE signs to the trainee. The Vscan employs a 1.7–3.8 MHz phased array probe and the Siemens P10 uses a 2–4 MHz phased array probe. Only one device was used throughout a CLUE exam. As the study tested real-time wireless guidance, no images were stored or reviewed off-line. All subjects were asymptomatic. Trainees, the sonographer, and echocardiography expert were blinded to their clinical histories. 

Screening accuracy and technical quality of transmitted CLUE images were compared to gold-standard echocardiograms that included lung apical imaging performed on a standard platform (Acuson Cypress, Siemens Healthcare). The gold-standard echocardiograms were obtained by a registered sonographer blinded to the CLUE results according to standard guidelines [8] and was interpreted by an expert echocardiographer in a blinded and randomized fashion. LVSD was defined as an interpreted ejection fraction <40% using the interpreter's discretion of all available methods including nonquantitative expert estimation, the biplane method of discs (modified Simpson's rule), or fractional shortening. LAE was defined by an anteroposterior LA diameter >4.0 cm or LA volume index >28 mL/m^2^ measured using the area-length method [8]. Interpretation of the eCVP and ULC+ findings on the echocardiogram used the same method as the PUD. All images were assigned a quality score: 0 (no image), 1 (only motion detected; off-axis), 2 (“suboptimal,” poor delineation of structures), 3 (“adequate” for diagnosis of particular sign), or 4 (“optimal,” good delineation of all structures equivalent to idealized standard echocardiographic view). Views with scores >2 were considered technically adequate.

The diagnostic sensitivity, specificity, positive and negative predictive values, and accuracy were derived for the CLUE diagnostic criteria for LVSD, LAE, ULC+, and eCVP by comparing the interpretation of technically adequate CLUE views with the results of LVSD, LAE, ULC+, and eCVP from the reference standard echocardiogram. The Scripps Institutional Review Board approved the study.

## 3. Results

Successful transmission and guidance occurred in 27/27 patients among the three trainees: trainee 1: 10 patients; trainee 2: 8 patients; trainee 3: 9 patients. All of the 27 expert-guided CLUEs were successfully completed in less than 5 minutes. Two transmissions (7%) were dropped and reinitiated. Guided novice versus sonographer imaging yielded technically adequate CLUE views in 122/135 (90%) versus 130/135 (96%), respectively (*P* = 0.05). CLUE had a combined sensitivity, specificity, and accuracy of 0.67, 0.96, and 0.90 for echo thresholds. Technical adequacy (%) and accuracy for each abnormality were LVSD (85%, 0.93, *n* = 5), LAE (89%, 0.74, *n* = 16), ULC+ (100%, 0.94, *n* = 5), and eCVP (78%, 0.91, *n* = 1) (see Table 2).

## 4. Discussion

This feasibility study demonstrated that novices could perform a specific 4-view CLUE appropriate for PUDs, when guided in real time by an expert using wireless transmission. In spite of lesser technical quality than standard echocardiographic views, transmitted images provided substantial accuracy as a bedside examination. In the ASE REWARD study [1], volunteer physicians and sonographers screened 1030 subjects in India with PUD, then uploaded the images to a “cloud” where they were accessed and reviewed by expert physicians in other countries. However, this study required expert sonographers and had a median time interval of 12 hours and 11 minutes prior to image interpretations, which is inadequate for emergency application. Instead, our novel diagnostic and teaching method combines a simplified cardiac imaging protocol with inexpensive technologies and could facilitate the use of ultrasound for immediate off-site bedside medical decision making and triaging of out-of-hospital patients with common presentations of chest pain, unexplained dyspnea, or hypotension. For instance, in the field or the ambulance ride to the hospital, emergency medical personnel could perform this diagnostic technique with remote guidance by experts such as emergency physicians or cardiologists trained in the CLUE. LVSD has been shown to occur in many acute disease states including acute coronary syndromes, septic shock, and acute heart failure [9]. LAE has also been shown to be a marker for the presence of significant cardiac findings on echocardiography [10]. ULC+ can occur in patients with acute pulmonary edema or interstitial disease [11]. Lastly, evaluating the inferior vena cava for eCVP can be used in the assessment of different types of shock (septic, cardiogenic, and hypovolemic) as well as in cardiac tamponade and pulmonary embolism. Earlier diagnosis of these time-sensitive and critical conditions may lead to better patient outcomes through earlier management decisions. 

Limitations of these observations are as follows. The body mass indexes of the patients, which can affect image acquisition, were not measured. However, patients were typical of those seen in outpatient cardiology practice and none of the 27 patients were “morbidly obese” by subjective estimates. There was inadequate power to determine intertrainee differences due to the small number of pathologies and patients. By focusing on the average technical adequacy score over the training period, our observations likely underestimated the trainee's ability by the end of the study. Although this study showed that real time remote guidance of a cardiac examination performed by novices using a PUD is feasible in the outpatient setting and its brevity (<5 minutes) is clinically practical for emergency situations, further and larger studies incorporating additional signs such as pericardial effusion or right ventricular dilation are needed to apply this diagnostic and teaching method in the out-of-hospital emergency setting. 

## Figures and Tables

**Table 1:** Components of the CLUE Protocol.

Signs	Probe site	Diagnostic targets	Normal	Abnormal
LVSD	Parasternal long axis	Distance from the anterior leaflet of the mitral valve to the septum during diastole	≤1 cm	>1 cm
LAE	Parasternal long axis	Anteroposterior diameter of the LA versus overlying aorta throughout the cardiac cycle	Aorta > LA	LA > Aorta
ULC+	Bilateral lung apices	Number of lung comets in either lung apex	<3	≥3
eCVP	Subcostal	Percent luminal diameter reduction of IVC with respiration, without forced “sniffing”	>50%	≤50%

**Table 2:** Comparison of technically adequate CLUE views and diagnostic accuracy for each abnormality between guided-novice PUD and gold-standard echocardiogram.

Signs	(*n*)	Novice quality (% tech. adequate)	Echo quality (% tech. adequate)	Sens. (%)	Spec. (%)	PPV (%)	NPV (%)	ACC. (%)
LVSD	5	3.1 ± 0.8 (85)	3.7 ± 0.6 (96)	80	95	80	95	93
LAE	16	3.1 ± 0.7 (89)	3.7 ± 0.6 (95)	69	82	85	64	74
ULC+	5	3.2 ± 0.4 (100)	3.9 ± 0.4 (100)	40	100	100	94	94
eCVP	1	3.1 ± 1.0 (78)	3.4 ± 0.7 (89)	100	96	96	100	96

Total	27	3.2 ± 0.7 (90)	3.7 ± 0.5 (96)	67	96	82	92	90

*n*: number of abnormality; quality scores reported as mean ± SD; Sens.: sensitivity; Spec.: specificity, PPV: positive predictive value; NPV: negative predictive value; ACC.: accuracy.
